# Distant Mesenchymal Progenitors Contribute to Skin Wound Healing and Produce Collagen: Evidence from a Murine Fetal Microchimerism Model

**DOI:** 10.1371/journal.pone.0062662

**Published:** 2013-05-01

**Authors:** Elke Seppanen, Edwige Roy, Rebecca Ellis, George Bou-Gharios, Nicholas M. Fisk, Kiarash Khosrotehrani

**Affiliations:** 1 The University of Queensland, UQ Centre for Clinical Research, Herston Campus, Brisbane, Australia; 2 Kennedy Institute of Rheumatology, University of Oxford, Oxford, United Kingdom; 3 Centre for Advanced Prenatal Care, Royal Brisbane and Women’s Hospital, Herston, Australia; University Hospital Hamburg-Eppendorf, Germany

## Abstract

The contribution of distant and/or bone marrow-derived endogenous mesenchymal stem cells (MSC) to skin wounds is controversial. Bone marrow transplantation experiments employed to address this have been largely confounded by radiation-resistant host-derived MSC populations. Gestationally-acquired fetal MSC are known to engraft in maternal bone marrow in all pregnancies and persist for decades. These fetal cells home to damaged maternal tissues, mirroring endogenous stem cell behavior. We used fetal microchimerism as a tool to investigate the natural homing and engraftment of distant MSC to skin wounds. Post-partum wild-type mothers that had delivered transgenic pups expressing luciferase under the collagen type I-promoter were wounded. *In vivo* bioluminescence imaging (BLI) was then used to track recruitment of fetal cells expressing this mesenchymal marker over 14 days of healing. Fetal cells were detected in 9/43 animals using BLI (Fisher exact p = 0.01 versus 1/43 controls). These collagen type I-expressing fetal cells were specifically recruited to maternal wounds in the initial phases of healing, peaking on day 1 (n = 43, p<0.01). This was confirmed by detection of Y-chromosome+ve fetal cells that displayed fibroblast-like morphology. Histological analyses of day 7 wounds revealed vimentin-expressing fetal cells in dermal tissue. Our results demonstrate the participation of distant mesenchymal cells in skin wounds. These data imply that endogenous MSC populations are likely recruited from bone marrow to wounds to participate in healing.

## Introduction

Wound healing is a well-orchestrated process that can be divided into three overlapping phases, beginning with the inflammatory, followed by the proliferative and concluding with the remodelling phase [1]. Several cell types of mesenchymal origin have been implicated in these processes. Fibroblasts, fibrocytes and myofibroblasts play a critical role in both early and late phases where they contribute to the wound contraction, collagen deposition and finally fibrosis [2], [3].

Researchers have been attracted to the concept of delivering these cells therapeutically during wound healing to improve healing speed and quality. Mesenchymal stem cell therapy (MSC) has been one of the candidate strategies with the idea that this precursor population would integrate and differentiate into the above mentioned cell types. Despite benefits largely mediated through paracrine mechanisms, engraftment of transplanted MSC in the wound area is usually poor [4], [5]. This raises the question of the involvement of endogenous mesenchymal-lineage cells from distant sites like bone marrow to wounds, which has direct clinical implications, specifically addressing the appropriate source and route of administration of MSC for therapy.

One of the principal reasons bone marrow-derived mesenchymal cell contribution to wounds is controversial, arises from their complex phenotype and resultant absence of distinguishing cell surface markers with which to discriminate MSC *in vivo*. Consequently, their *in vivo* tracking has proven challenging. Bone marrow chimeras generated to address this question of source have produced conflicting data, because of radiation-resistant host-derived MSC [6], [7], [8], [9], [10]. Indeed, after bone marrow transplantation, bone marrow MSC were mostly recipient-derived and rarely replaced by the transplanted marrow cells [11], [12]. Here we propose to overcome this limitation of experimentally-induced bone marrow transplantation using a natural transplantation model of feto-maternal microchimerism that allows long term engraftment of chimeric MSC in maternal bone marrow in every pregnancy [13].

Feto-maternal microchimerism refers to the traffic of genetically-distinct fetal cells into the maternal circulation during pregnancy. Small numbers of these fetal microchimeric cells (FMC) engraft long-term [14], [15]. FMC have been implicated in maternal tissue repair since they selectively home to and integrate into sites of maternal tissue damage [16], [17], [18], often adopting site-appropriate phenotypes [15], [19]. The transfer and persistence of diverse fetal stem and progenitor cells has been conclusively established and the phenotypes of FMC include hematopoietic [14], [20], endothelial [16], [17], [21], [22], mesenchymal [13], [23] and even neuronal [24], [25] stem and progenitor cells [26].

Chimeric mesenchymal stem cells (MSC) of fetal origin were first identified in the blood of a woman undergoing elective termination in her first trimester of pregnancy [23]. In a subsequent study, fetal MSC were detected in bone marrow stromal cultures from 100% of post-partum women, indicating that persistence of fetal MSC is likely to be ubiquitous in every pregnancy. These Y-chromosome+ve fetal cells expressed markers consistent with an MSC phenotype, and like their neighbouring maternal MSC, also displayed both osteogenic and adipogenic differentiation [13]. These data established that fetal MSC or a more primitive mesodermal progenitor are acquired through pregnancy and remain engrafted long term in maternal marrow. This offers an alternative paradigm to study the potential recruitment and activity of endogenous stem and progenitor cells to sites of tissue injury. Since we know FMC are recruited to maternal skin wounds [22], [27], we employed two microchimerism models to address the involvement of distant mesenchymal lineage cells in wounds. Using different transgenic strategies to track fetal cells, we showed the specific homing and mesenchymal potential of FMC. These data support a therapeutic model where a systemic delivery of mesenchymal cells could participate in wound healing and collagen deposition.

## Methods

### Animals

Wild-type (WT) C57BL/6 females (Animal Resources Centre, Western Australia, Australia) were serially bred with males hemizygous for the luciferase reporter, driven under the collagen type 1 alpha 2 chain promoter (Col1a2) developed by one of us (GBG) [28]. In this microchimerism model [29] subsequently referred to as the Col1-Luc model, only collagen type I producing FMC express luciferase, allowing us to track the natural recruitment of fetal MSC to maternal wounds. In parallel, WT females were serially bred with males hemizygous for the enhanced green fluorescent protein (GFP) inserted into the ROSA26 locus on a C57BL/6 background B6.Tg(Gt(ROSA)26sor-EGFP)IIAble (B6ROSAeGFP) (JAX®, Maine, USA). In this model, referred to as the GFP model, FMC were labeled with GFP and thus not discriminated by their phenotype, allowing us to calculate the relative proportion of these mesenchymal FMC amongst other sub-types. After each delivery we ensured that at least one luciferase+ve or GFP+ve pup could be identified in litters. WT females serially-bred with WT males served as technical controls. This control group (denoted as WT FMC throughout) allowed us to eliminate potential pregnancy-associated effects and monitor background signal produced by *in vivo* bioluminescence imaging. WT virgin females were also used as technical controls. All mice were treated in accordance with institutional guidelines and ethical approvals for the care of experimental animals. The protocols were approved by the University of Queensland Animal Ethics Committee (permit number: UQCCR 1016/08/NHMRC). Wounding was performed under inhalation anesthesia-isofluorane and all efforts were made to minimize suffering.

### Dorsal Excisional Wound Model

Dorsal hair was removed completely, first with clippers and then via the application of Veet® depilatory cream. Under anesthetic, 2× full-thickness excisional wounds were generated down to the panniculus carnosus with a 6 mm sterile punch biopsy (Stiefel, Germany). Wounds were left open and animals sacrificed at defined time-points post-wounding.

### In vivo Bioluminescence Imaging: Image Capture

Wounded mice were injected with 200 µl (150 mg/kg) D-luciferin substrate (Caliper Life Sciences Inc, MA, USA). After 10 mins, mice were anesthetised (3% iso-flurane) and placed dorsally exposed in a Xenogen IVIS® Spectrum (Caliper Life Sciences Inc, MA, USA). Images were acquired and light was captured using a CCD camera at −95°C. Acquisition parameters were as follows; 5 mins exposure, F/stop 1, binning 4, field of view B. Each image acquisition included a control versus experimental animal. Animals were imaged before wounding (but after hair removal) and regularly from 1–14 days post wounding.

### In vivo Bioluminescence Imaging: Image Analysis

Living image software (Caliper Life Sciences Inc, MA, USA) was used for bioluminescence analyses. Equal-sized regions of interest (ROI) were drawn around each wound and the rate of light emitted was calculated and represented as total flux (photons/sec). Data were plotted against time.

### Tissue Processing

Dissected wounds were fixed for 2 hrs in 4% PFA and either infused with 10% sucrose before cryo-embedding. For cryo-mounting, sucrose-infused wounds were mounted in optimal cutting medium (O.C.T) and frozen in a bath of dry ice and ethanol. 7 µm sections were subsequently cut on a cryo-stat for immuno-histochemistry studies.

### Immuno-histochemistry

For staining of specific antigens, cryo-sections were permeabilized in 0.5% Triton-x-100 before blocking with 10% normal goat serum. Primary antibodies included: 1 in 500 rabbit anti-GFP (Abcam, Cambridge, UK) or 1 in 100 chicken anti-GFP (Invitrogen, CA, USA), 1 in 100 rat anti-mouse CD45 (Becton Dickinson, NJ, USA), 1 in 200 rabbit anti-mouse alpha smooth muscle actin (α-sma), 1 in 200 chicken anti-mouse vimentin (Abcam, Cambridge, UK) and 1 in 50 rat anti mouse CD31 (Becton Dickinson, NJ, USA). Secondary antibodies conjugated with Alexa-fluor 568 or 488 or 647 (Invitrogen, CA, USA) were used for fluorescence detection. Nuclear staining was revealed in specimens mounted with ProLong® Gold mounting media containing DAPI (Invitrogen). Images were captured using an Axio Imager M1 (Zeiss, Germany) or a LSM 710 confocal microscope (Zeiss, Germany).

### Fluorescence in situ Hybridization (FISH)

To reveal male fetal cells amongst maternal cells, Y-chromosome FISH was performed as described previously [30]. Briefly, wound cryo-sections were rehydrated in distilled water before pretreating with 0.2M HCl for 20 mins. Slides were then incubated at 80°C in 2×SSC for 20 mins and then dipped in distilled water and washed in 2× SSC/0.05% Tween-20 for 5 min. Sections were next digested at 37°C for 7 mins with TEN buffer containing 0.05 mg/ml Proteinase K (USB corporation, OH, USA). Slides were subsequently washed in 2× SSC.0.05% Tween-20 for 5 mins and fixed in 4% formaldehyde for 10 mins. Again, slides were washed in 2× SSC/0.05% Tween-20 and dehydrated through a series of ethanol washes (70%, 90% and 100%) for 2 min each. Mouse IDetect™ Y chromosome probe labeled with IDYE™495 (ID Labs_Inc_™, Ontario, Canada) diluted in hybridization buffer was applied to sections. Coverslips were sealed with rubber cement and DNA was denatured at 90°C for 10 mins before an overnight incubation at 42°C. Coverslips were removed and slides were washed in 2× SSC for 10 min at 42°C followed by 2×5 min washes in 2× SSC/50% formamide at 42°C. Excess formamide was removed by a 5 min wash with 2× SSC. Nuclear staining was revealed in specimens mounted with ProLong® Gold mounting media containing DAPI (Invitrogen). Images were captured using an Axio Imager M1 (Zeiss, Germany).

### Scoring and Quantification

To eliminate bias, all slides for Masson’s trichrome quantification and FMC engraftment were scored blind to the gestational history of the animals. For detection of GFP+ve or FMC, antibody-labeled slides were first scanned using a dual GFP/dsRED filter and cells emitting only green-specific fluorescence were selected. To include these as FMC, the selected cells also had to contain a nucleus, have a regular cell membrane and emit GFP fluorescence at comparable levels to mononuclear cells from control B6ROSAeGFP animals. Finally, to be counted as true FMC, cells had to have homogenous GFP staining under high magnification (40× objective).

### Statistical Analysis

Analyses were performed using GraphPad Prism v5c software. Data generated by longitudinal studies were analyzed by two-way ANOVA. Normally-distributed data were evaluated by standard t-tests while non-parametric tests, such as Mann-Whitney were used on skewed data and Fisher exact testing was used for categorical analyses. A p value<0.05 was considered significant.

## Results

### Generation of the col1a2 Model: Breeding Strategy Ensures Transfer of Labeled Mesenchymal-lineage FMC

**Figure 1:**
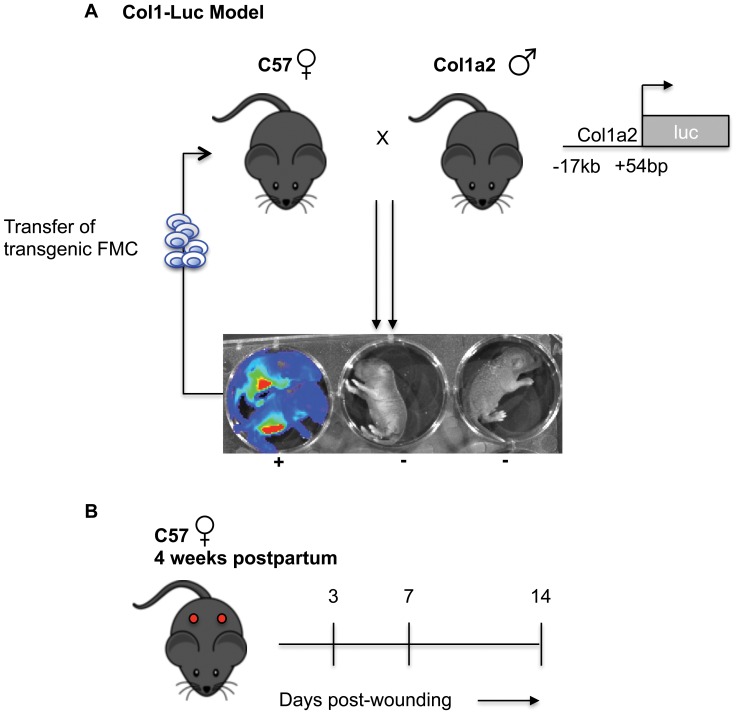
Generation of Col1a2 model of mesenchymal microchimerism. (**A**) Breeding strategy: WT females were mated serially with hemizygous col1a2 transgenic males that produce luciferase in collagen type I expressing cells. Using this strategy, ca. 50% of the progeny and therefore pregnancy-acquired FMC were labeled by the paternally-inherited transgene (Experimental group). WT females mated only with WT males served as controls. (**B**) Injury Model: Dorsal excisional wounds were generated on all post-partum WT females 4 weeks after their last delivery. Serial BLI imaging was then performed on all animals and tissues were collected at days 3, 7 and 14 post-wounding.

Since collagen type I production by MSC and their progeny is essential for normal wound healing [3], [6], we employed the Col1a2 transgenic mouse line in a wound healing experiment. Serial bioluminescence imaging (BLI) of Col1a2 transgenic mice along wound healing confirmed previously-reported kinetics of collagen expression (data not shown) [6]. After establishing this activity of the Col1a2 promoter in our hands, we used this transgenic line to generate a model of mesenchymal microchimerism (Col1-Luc model). Experimental WT virgin females were mated serially to Col1a2 males (Figure 1A). Using this strategy, only the fetal collagen type I expressing cells acquired by mothers expressed luciferase and thus could be detected by BLI. WT virgin females serially mated to WT males served as controls (denoted throughout as WT FMC). Experimental mothers (designated as Col1a2 FMC) had at least 2 confirmed pregnancies and the delivery of luciferase+ve pups and male pups was confirmed each time. Four weeks after delivery of the final litter, two full thickness dorsal wounds were generated on all post-partum females (both control and experimental). Animals were then subjected to serial BLI imaging and wounds were collected at days 3, 7 and 14 post-wounding (Figure 1B).

### BLI Signal in Post-partum Females Reflects the Rapid Recruitment of Collagen Type 1 Expressing FMC and Therefore Endogenous MSC to Wounds

**Figure 2:**
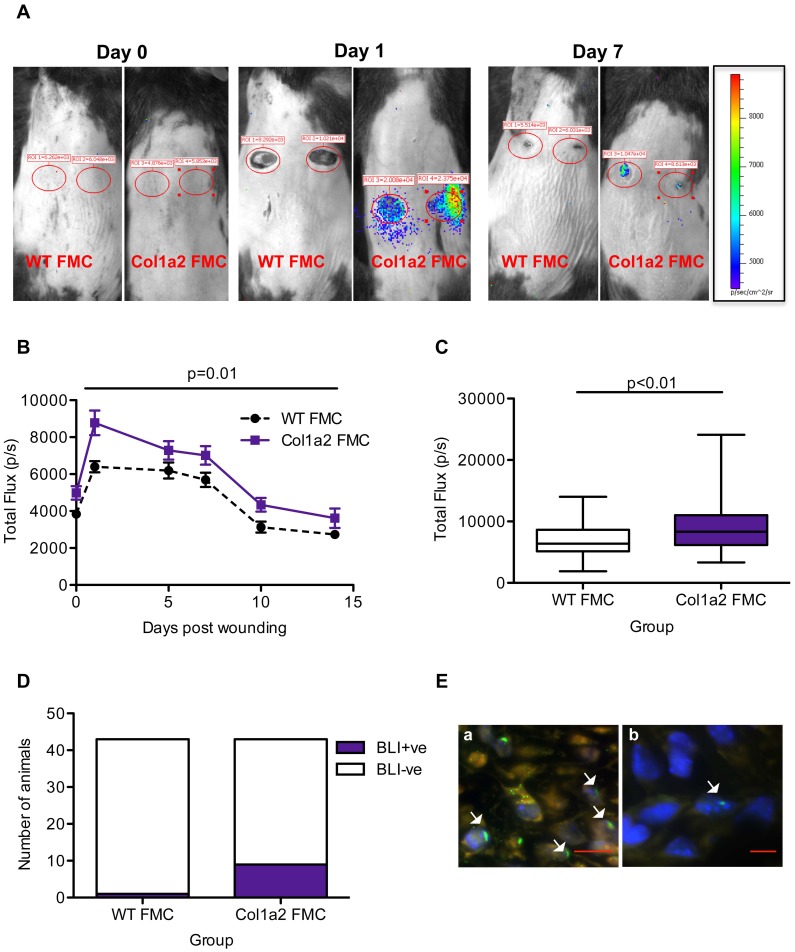
Collagen producing FMC are recruited to maternal skin wounds as early as day 1. (**A**) Representative BLI images across wound healing. All animals were post-partum WT females but those labeled WT FMC had produced only WT fetuses whereas those labeled Col1a2 FMC had produced Col1a2 transgenic fetuses. Red circles = ROI kept constant over time for comparison of measurements. Signal was absent in unwounded skin (day 0). (**B**) BLI signal, represented as total flux (photons/s), was tracked over the course of wound healing. Signal was always higher in Col1a2 FMC animals (receiving transgenic FMC) (ANOVA on D7 or D14 cohorts p = 0.01). Maximal signal was observed day 1 after wounding. (**C**) Box and whisker plots represent the median and range of signal produced from wound regions at day 1. Signal was more intense in WT animals receiving transgenic FMC (p<0.01). (**D**) Animals with high BLI signal (>99^th^ percentile of background). In the Col1a2 FMC group, 9/43 animals compared to 1/43 animal in the WT FMC group had high BLI signal (Fisher exact p = 0.01). (**E**) Microchimerism was confirmed in wound tissue from high BLI animals by the detection of Y-chromosome+ve fetal cells. (**E-a**) Male wound positive control. White arrows point to examples of nuclei with Y-chromosome signal. (**E-b**) Representative image of a single Y-chromosome+ve fetal cell (white arrow) found amongst maternal dermal cells in the wound center. Scale bars represent 10 µm.

To track the recruitment of collagen type I expressing FMC to maternal wounds, serial BLI was performed. Wounds are known to result in potential return of false signal by reflection of light on a humid surface [31], [32]. Therefore, great care was taken in selecting age and pregnancy history matched controls that were imaged in parallel to experimental animals during each acquisition. Here, WT mothers that had only delivered WT pups served as technical controls for both imaging limitations and possible side-effects from pregnancy and age. As expected, the level of background bioluminescence in this control group peaked in the days after wounding and returned to baseline levels by day 10 upon wound closure. However, in some animals that had delivered transgenic pups, a specific signal from wounds and not uninjured skin could be seen, reflecting the presence of collagen type I expressing FMC (Figure 2A). Mapping the kinetics of the signal captured from wounds revealed a rapid recruitment of collagen type I expressing FMC as early as day 1 post wounding (Figure 2B). The signal in mothers that had delivered transgenic pups was always above the background signal captured from equivalent regions of interest (ROI) in control animals (ANOVA with day 7 or day 14 cohort p = 0.01). When individual time-points were considered in post-hoc testing, the difference was significant from control animals during early wound repair (Mann Whitney p<0.01) (Figure 2C) but not as wounds healed.

Because these data represent the average signal produced from all mice, and since FMC presence in mice is stochastic [33], we performed a sub-analysis on animals with positive BLI signal. For animals to be considered BLI+ve, the signal emitted from wounds had to be greater than the 99^th^ percentile of background luminescence observed in controls. Among WT females that gave birth to transgenic offspring, 9/43 animals had high signal compared to 1/43 animals in the group that delivered WT offspring (Fisher exact p = 0.01) (Figure 2D).

We used FISH as a further technique to confirm the presence of FMC. Y-chromosome was detected robustly in all cells of wound specimens from male mice (Figure 2E-a) but never in virgin control specimens, validating the technique. Y-chromosome+ve cells were detected in wounds from animals considered BLI+ve, with a median of 5 Y-chromosome+ve fetal cells/section (Figure 2E-b). Since these FMC were clearly transcribing collagen, we explored a potential relationship between overall collagen deposition in late wounds (day 14) as measured by the surface of Masson’s trichrome staining and BLI signal (day 1). However, Spearman analysis revealed a lack of correlation. We also tested whether the presence of collagen producing FMC, as indicated by high BLI signal at day 1, influenced wound closure. Wound closure was similar in animals with high BLI signal compared to those with the lowest signal (day 1), suggesting that collagen producing FMC do not influence wound closure (Figure S1). Overall, these data suggest the recruitment of a population of fetal mesenchymal cells into wounds that activate the collagen type 1 promoter.

### FMC in Maternal Wounds Display Diverse Phenotypes

**Figure 3:**
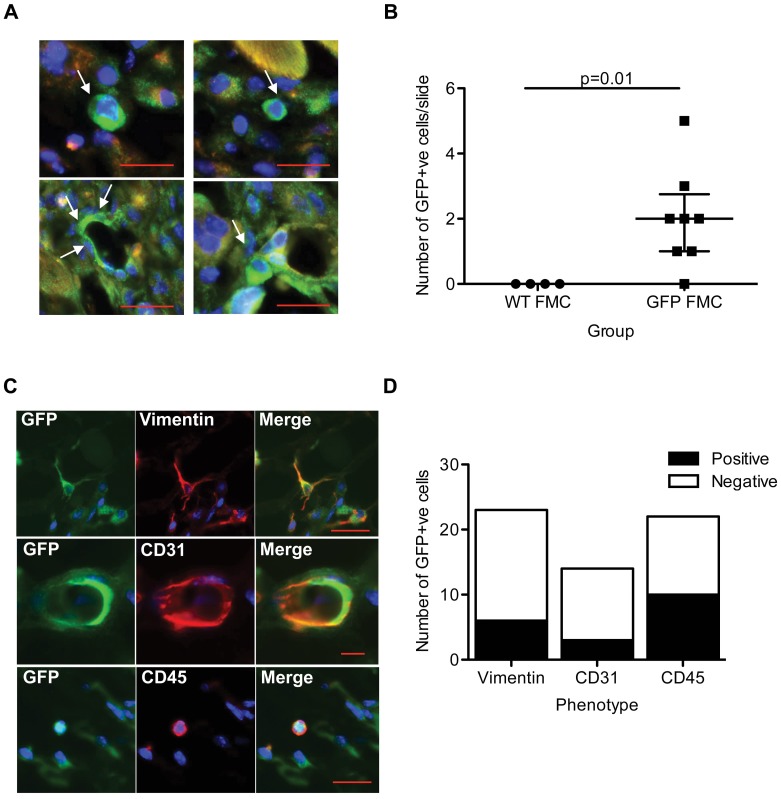
FMC engraft maternal skin wounds. (**A**) GFP model. In parallel to the col1a2 model, post-partum WT animals that received either GFP+ve FMC (GFP FMC) or WT FMC (WT FMC) were wounded. Panel represents GFP+ve FMC found in day 7 wounds. These GFP+ve FMC (white arrows) displayed diverse morphologies. (**B**) Blinded scanning of slides 7 days after wounding revealed true GFP+ve FMC found only in the GFP FMC group (Median+Interquartile range, Mann-Whitney p = 0.01). (**C**) Phenotypes of GFP+ve FMC. Representative GFP+ve FMC found in wounds co-labeled with either vimentin, CD31 or CD45. All nuclei are stained with DAPI (blue). Scale bars represent 20 µm. All images were captured with a Zeiss Axio Imager M1 fluorescent microscope. (**D**) Graphical representation of diverse FMC phenotypes found in maternal wounds. Discussion.

To investigate thoroughly the phenotypes of the mesenchymal FMC engrafted in maternal wounds *in situ*, we generated an additional microchimerism model. Here, post-partum WT mothers that had produced GFP+ve pups after serial breeding with B6ROSAeGFP males underwent dorsal skin wounding. Again, post-partum WT mothers that had only mated with WT males served as controls (WT FMC group). Wound sections were stained with anti-GFP antibody and scored blind to the gestational history. GFP+ve FMC (Figure 3A) were found in wounds from 88% (7/8) of animals that had delivered GFP+ve pups, but never found in controls (0/4) (Fischer Exact p = 0.01) with a median of 2 fetal cells per section (Mann Whitney p = 0.01) (Figure 3B). In addition, FMC were never detected in uninjured skin prior to wounding (0/4), suggesting their recruitment only in response to wounding. To phenotype the FMC in maternal wounds, sections were co-stained with a panel of markers (Figure 3C). In accordance with our previous investigation of FMC sub-types in maternal wounds, we detected both hematopoietic and endothelial FMC sub-types. GFP+ve FMC co-expressing CD31 appeared to connect to the maternal vasculature and represented ∼27% of total FMC. Similarly, CD45+ve FMC were found more frequently (10/22), equating to∼46% of total GFP+ve FMC in day 7 wounds (Figure 3D). As per our initial publication, we found that ∼20% of these fetal cells were neither hematopoietic nor endothelial [22]. GFP+ve FMC co-expressing vimentin (6/23) were found, representing ∼26% of total GFP+ve cells. Although less frequent, GFP+ve FMC co-expressing α-sma were also identified (2/30) (Figure S2). In addition, we performed triple staining with GFP, CD45 and vimentin to examine whether FMC in wounds could indeed be fibrocytes [34]. Although we could find GFP+ve FMC expressing either CD45 or vimentin (at proportions similar to those described above), we could only find a single example of a GFP+ve CD45+ve vimentin+ve FMC (data not shown) suggesting that fibrocytes are not the main type of FMC.

The recruitment of endogenous mesenchymal lineage cells from distal sites to wounds is controversial, largely due to their unreliable *in vivo* traceability and absence of engraftment after classical bone marrow transplantation. Feto-maternal microchimerism offers a complementary paradigm to investigate endogenous stem cell homing and engraftment, particularly of populations with complex phenotypes. By employing two models of fetal microchimerism, we demonstrated that fetal cells persisting beyond pregnancy are recruited to maternal wounds and include a sub-population with mesenchymal capacity. In the Col1-Luc model, *in vivo* tracking of collagen type I-producing FMC during wound healing revealed their rapid recruitment to wounds from as early as day 1. The production of collagen by these fetal cells demonstrated both their mesenchymal capacity and functionality, albeit without influencing wound healing. In another paradigm (GFP model), where FMC were not discriminated by their collagen production, a sub-population of FMC were found that had a mesenchymal phenotype based on their expression of vimentin and α-sma. Therefore, through an alternative investigative paradigm, we have established that mesenchymal lineage cells can indeed be recruited from distant sites to acute wounds.

It is well known that long-lived FMC selectively home to, and engraft at, sites of maternal tissue damage [17], [18], [22], [25], [35]. We have shown previously, the utility of murine microchimerism models for tracking endogenous stem cells *in vivo*, particularly in response to different skin injuries [16], [17], [22]. Phenotyping FMC recruited to maternal skin wounds has demonstrated hematopoietic and endothelial capacity [22], while their mesenchymal capacity has been demonstrated in inflamed appendices and injured kidneys [29], [36].

While this mesenchymal capacity by FMC is well established, it is still not known when FMC acquire their mesenchymal nature on their journey from fetus to mother. Indeed fetal MSCs have been found to niche in maternal bone marrow [13] for decades but they have also been found in the maternal circulation during the first trimester of pregnancy [37]. These observations would support a theory whereby fetal cells acquired by mothers already have an MSC phenotype. Indeed, diverse fetal cell types (that could include MSC) are acquired stochastically as a result of feto-maternal hemorrhage [23]. FMC captured as they enter the maternal circulation during pregnancy are known to display diverse surface markers and genetic signatures that confer specific cell types [38], [39], supporting this hypothesis. However, one cannot exclude an alternative hypothesis; that FMC are the result of a primitive fetal stem cell population acquired during early pregnancy. Such a primitive fetal stem cell could then give rise to multi/uni- potent fetal progenitors that persist and differentiate appropriately down the meso-, ecto- and endo- dermal lineages in maternal tissues [21], [40], [41]. These could of course include fetal MSC. Regardless of whether or not fetal MSC are transferred directly from fetal to maternal circulation, there is substantial evidence for the presence of fetal cells in maternal blood and tissues (both human and murine) with the propensity to differentiate down mesenchymal lineages [13], [23], [29], [36].

There are no reliable markers to track endogenous MSC *in vivo* as most are shared with hematopoietic (e.g. Sca1) and endothelial cells (e.g. CD105). Thus using a traditional technique for endogenous stem cell tracing like combinatorial flow cytometry is not applicable to robustly delineating MSC *in vivo* [42], [43]. Using a microchimerism model that exploited the production of collagen I by MSC [6], [44], we traced mesenchymal cells *in vivo* for the first time in skin. The BLI signal observed in wounds from mothers that had delivered col1a2 transgenic pups reflected rapid collagen I production solely by these FMC, suggesting their contribution to repair at a time point (day 1) that was earlier than expected. Given that some transcriptional regulation is shared between collagen III and collagen I [45], [46], it is plausible to consider that this discrepancy in timing could also reflect simultaneous activation of the collagen III transcriptional machinery in FMC. Regardless, this collagen promoter activity by FMC revealed both their contribution to wound repair and mesenchymal capacity, which was confirmed in the GFP model where ∼26% of total FMC expressed the mesenchymal marker, vimentin and to a lesser extent, α-sma. This is highly consistent with our previous study of FMC in maternal wounds where we reported ∼80% of FMC to be either hematopoietic (CD45+ve) or endothelial [22], leaving ∼20% undefined that we have now shown to be mesenchymal and rarely dual CD45+ve vimentin+ve.

Collectively, these data also suggest the likely bone marrow origin of mesenchymal cells in wounds. Although results are controversial, bone marrow transplantation experiments have suggested that collagen type I producing cells in skin wounds are dermally resident [6], [8]. Previous studies have confirmed that extremely-rare FMC with mesenchymal capacity reside in appropriate bone marrow-MSC niches in post-partum women, supporting our claim that the fetal mesenchymal cells recruited to skin wounds are bone marrow-derived [13]. It could be argued that fetal cells were already in the skin before wounding. However, in animals that had produced GFP+ve pups, we could not detect GFP+ve FMC in uninjured skin prior to wounding. Previous data has also established that FMC are rarely found in uninjured tissues [41], [47], [48]. Additionally, FMC have also been found to implant in acellular material driving angiogenesis, confirming their ability to be recruited rather than proliferate locally [22]. Moreover, GFP+ve FMC found in the granulation tissue in our study were identified as either individual single cells or pairs of cells amongst maternal cells, as opposed to clusters of cells that would otherwise suggest proliferation. This supports our interpretation of the BLI data in the Col1-Luc model that the signal observed in maternal wounds reflects recruited fetal mesenchymal cells. Our data are further supported by bone marrow transplantation of adherent cell populations, where donor MSC were presumably transferred. Bone marrow-derived spindle-shaped CD45 negative donor cells were found in wound tissue from repopulated recipients [7], [44] and confirmed as expressing collagen I, Thy-1 and α-sma [44].

Given the likely bone marrow origin of FMC, their collagen I production, and fibroblast morphology in wound tissue, we cannot exclude the possibility that the BLI data reflect the rapid recruitment of fetal fibrocytes [49], [50], [51]. This is especially since the maximal recruitment of collagen producing FMC to maternal wounds occurred on day 1, corresponding with the arrival of leukocytes [52], and not the day 7 peak of col1a2 promoter activity seen in dermal fibroblasts [6]. During normal wound healing, bone marrow-derived collagen I+ve CD45+ve fibrocytes are able to differentiate into contractile wound healing myofibroblasts [34]. However, we could find only one example of a GFP+ve FMC with a potential fibrocyte phenotype in day 7 wounds suggesting that while they may be recruited as fibrocytes they weren’t persisting as fibrocytes.

### Conclusion

Fetal microchimerism following gestation represents a natural model of stem cell transplantation, allowing the study of persistent fetal cells comprising different stem and progenitor populations. It avoids the confusion generated by experimentally-induced transplantation or adoptive transfer models, particularly for cell types such as MSC that do not engraft upon bone marrow transplantation. Using two microchimerism models, we have shown that endogenous mesenchymal lineage cells from distant sites are recruited to and engraft in wound tissue and produce collagen. These data contribute to the understanding of the role of endogenous stem cells in response to tissue injury.

## Supporting Information

Figure S1 **Overall collagen production in wounds is not correlated to BLI signal. (A)** Representative image of Masson’s trichrome staining in day 14 wounds. Blue staining signifies total collagen. Scale bars represent 100 µm. **(B)** Spearman correlation confirmed a lack of relationship between BLI signal at day 1 and overall collagen production (quantitated as amount of blue staining). (C) Wound closure (as measured by the percentage of day 1 wound area) was not different between animals with high BLI signal (n = 9 animals >99^th^ percentile of background) and low BLI signal (n = 8 animals with lowest BLI signal) at day 1.
(TIF)

Figure S2 **α-Sma+ve FMC confirm their mesenchymal capacity. (A)** GFP model. Representative GFP+ve FMC found in wound tissue co-labeled with α-sma (red). Nuclei are stained with DAPI (blue). Scale bars represent 20 µm. Images was captured with a Zeiss Axio Imager M1 fluorescent microscope.
(TIF)
