# Supplementary figures and images for: Distant Mesenchymal Progenitors Contribute to Skin Wound Healing and Produce Collagen: Evidence from a Murine Fetal Microchimerism Model
Source: PLoS One. 2013 May 1;8(5):e62662. doi: 10.1371/journal.pone.0062662 (PMC3641113; doi:10.1371/journal.pone.0062662)

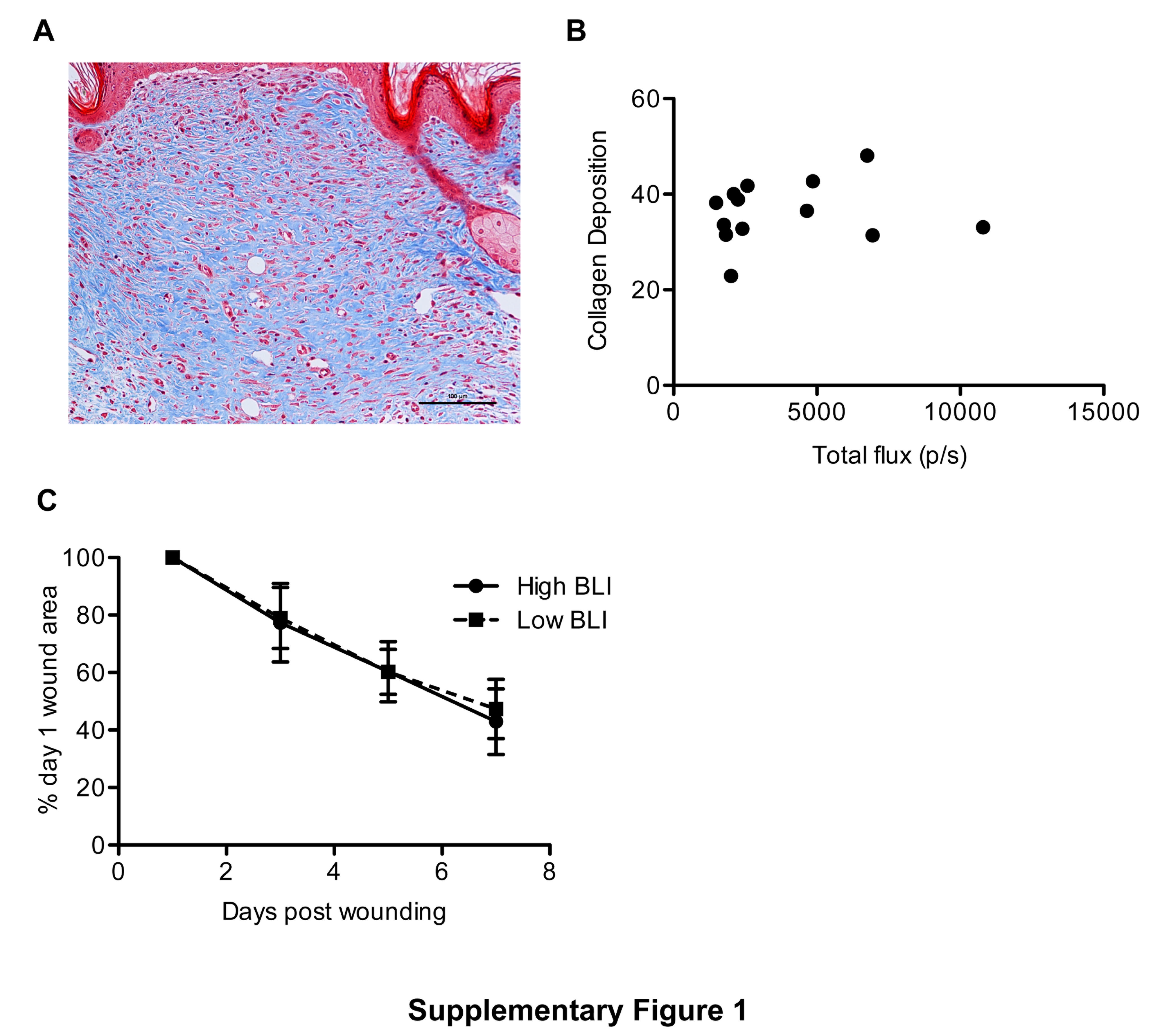

Supplement: Figure S1 — Overall collagen production in wounds is not correlated to BLI signal. (A) Representative image of Masson’s trichrome staining in day 14 wounds. Blue staining signifies total collagen. Scale bars represent 100 µm. (B) Spearman correlation confirmed a lack of relationship between BLI signal at day 1 and overall collagen production (quantitated as amount of blue staining). (C) Wound closure (as measured by the percentage of day 1 wound area) was not different between animals with high BLI signal (n = 9 animals >99th percentile of background) and low BLI signal (n = 8 animals with lowest BLI signal) at day 1. (TIF) [file pone.0062662.s001.tif]

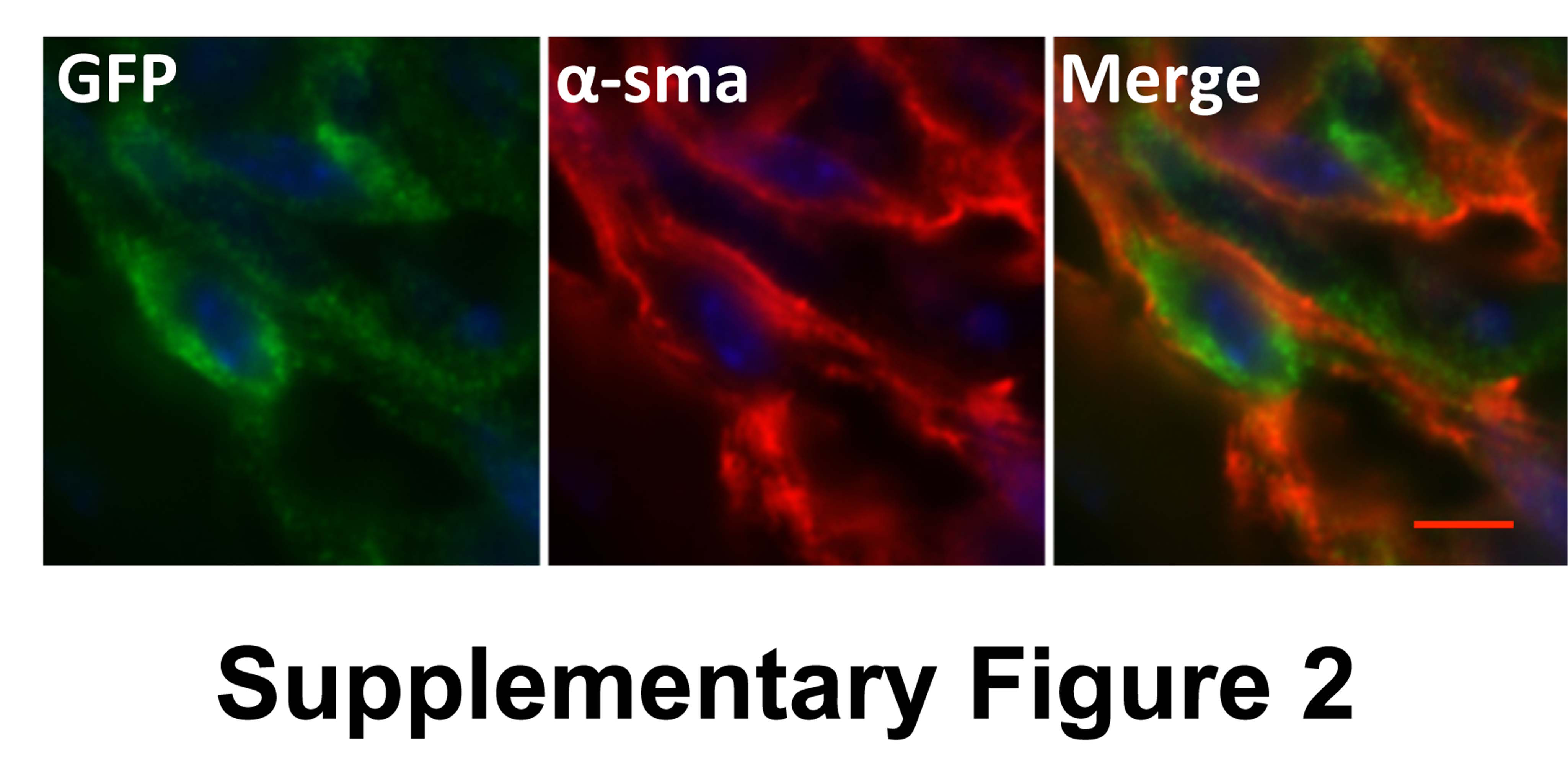

Supplement: Figure S2 — α-Sma+ve FMC confirm their mesenchymal capacity. (A) GFP model. Representative GFP+ve FMC found in wound tissue co-labeled with α-sma (red). Nuclei are stained with DAPI (blue). Scale bars represent 20 µm. Images was captured with a Zeiss Axio Imager M1 fluorescent microscope. (TIF) [file pone.0062662.s002.tif]
